# Closing the stroke care gap: a collaborative, self-sustainable telemedicine enabled model from rural Nepal

**DOI:** 10.3389/fstro.2026.1818548

**Published:** 2026-06-08

**Authors:** Khechar Nath Paudel, Mahesh Kumar Khanal, Lekhjung Thapa, Raju Paudel, Christoph Gumbinger, Apsara Hamal, Christine Tunkl

**Affiliations:** 1 Department of Internal Medicine, Province Hospital, Karnali Province, Surkhet, Nepal; 2 Ministry of Health, Lumbini Province, Dang, Nepal; 3 National Neuro Center, Kathmandu, Nepal; 4 Department of Neurology, Grande International Hospital, Kathmandu, Nepal; 5 Department of Neurology, University Hospital Heidelberg, Heidelberg, Germany; 6 Patan Academy of Health Sciences, Lalitpur, Nepal

**Keywords:** capacity building, low resource setting, stroke awareness, stroke care, telemedicine, telestroke

## Abstract

**Background:**

Stroke is a challenging global public health concern, disproportionately affecting people in rural communities. Stroke care is challenging in low and middle-income countries as it requires a coordinated multidisciplinary approach integrating pre-hospital recognition; acute stroke care, and long-term rehabilitation. Multiple barriers exist in LMICs: lack of community awareness; geographical and financial barriers; poor health systems; absence of standardized care pathways; and inadequate training among primary health care workers. Based on this background, this descriptive implementation-focused retrospective program evaluation describes the implementation and early impact of a collaborative, telemedicine supported multi-component health system intervention for stroke care in a rural government hospital in Nepal.

**Methods:**

A non-government organization “Nepal Stroke Project (NSP)” partnered with Province Hospital Surkhet (PHS), a community-based tertiary center in remote western Nepal strengthening the stroke care capacity in the region via formation of a multidisciplinary stroke team, infrastructure development and capacity strengthening. NSP experts also provided telemedicine supported clinical guidance to the local stroke team through free digital platform such as WhatsApp. The program evaluation was guided by the RE-AIM framework and interpreted through a health systems strengthening perspective.

**Results:**

Baseline assessment identified major system-level barriers, including the absence of a dedicated stroke pathway, thrombolysis services, stroke-specific infrastructure, and specialist support. Following implementation, annual stroke admissions increased from 154 to 178 cases per year, and 10 healthcare personnel were trained. Intravenous thrombolysis, previously unavailable, was successfully administered to two patients, supported by telemedicine-guided decision-making and subsequent ICU transfer. Over the implementation period, 20 stroke patients received telemedicine consultations, routine stroke pathway activation was achieved for thrombolysis cases, and NIHSS documentation improved from absent at baseline to approximately 50% of cases. Service readiness was further strengthened through establishment of two dedicated stroke beds and provision of essential monitoring equipment.

**Discussion:**

The collaborative model has a potential for sustainable impact by strengthening long term capacity building, and enabling the local team to deliver comprehensive stroke care independently. This implementation model highlights the importance of maximizing existing resources through task-shifting, integrating stroke care within existing health systems, and fostering local ownership to ensure sustainability.

## Introduction

In rural communities, the absence of timely and quality stroke care can transform a healthy working population into lives marked by disability or premature death. Stroke, or cerebrovascular accident (CVA), occurs when blood flow to a part of the brain is suddenly interrupted—either by a blockage (ischemic stroke) or a rupture (hemorrhagic stroke; Smith et al., 2015). Stroke care is a complex multidisciplinary process that covers the entire disease trajectory. The challenges of stoke care in low resource settings are limited awareness, financial barriers, weak health systems and inadequate emergency preparedness including lack of stroke units, neurologists, certified stroke hospitals, standardized protocols, and organized post-rehabilitation care (Pandian et al., 2020; Ferreira et al., 2024; Ghandehari, 2011). These gaps are particularly concerning, as stroke disproportionately impacts rural populations: 87.2% of global stroke deaths and 89.4% of global DALYs occur in low- and middle-income countries (LMICs; Feigin et al., 2024).

Nepal, a predominantly rural Himalayan nation in South Asia with mountainous terrain and dispersed settlements, has a low per capita GDP and significant challenges in equitable health care (Data Explorer, 2025). With an ethnically diverse population of 30 million people, and 60% of those living in rural areas (National Statistics Office, 2025), Nepal has a three tiered health care system, primary care provided by health posts and primary health care centers, secondary care through district hospitals and tertiary care through a few tertiary hospitals (UNFPA Nepal, 2020). There is a heterogenous mixture of public and private health care providers; and the National health insurance program though present has a very limited coverage [Ministry of Health and Population (MoHP), Government of Nepal, 2024]. The doctor-to-patient ratio in Nepal reflects a stark disparity, there are 1:850 in the Kathmandu Valley, but as low as 1:150,000 in many rural regions—significantly below the WHO's recommended minimum of 1:1,000 (World Health Organization, 2025). This imbalance is further compounded by limited availability of neurologists and stroke units, which remain largely concentrated in urban centers—leaving the tertiary centers of remote regions critically underserved. In Nepal, the estimated incidence of all types of stroke is 106.5 per 100,000 population, with stroke-related mortality reaching 74.1 per 100,000 and disability-adjusted life years (DALYs) burden of 1,454.1 per 100,000 (Venketasubramanian, 2025). Alarmingly, the proportion of haemorrhagic stroke in Nepal (26.79% of the total stroke; Paudel et al., 2023) is higher than in high-income countries, with death rates as high as 35.2 per 100,000 (Venketasubramanian, 2025). As a result, the nationwide thrombolysis rate—an important indicator of highly time dependent evident based intervention for acute stroke care which can even be delivered by non-specialist physician—remains below 2.5% (Paudel et al., 2023). Despite these realities, stroke care remains largely neglected by both government and other healthcare stakeholders. This situation is even worse, in Karnali province of Nepal, characterized by geographical and social inequalities, including high poverty, early marriage, higher than average maternal, and limited health workforce capacity [Ministry of Health and Population (MoHP), Government of Nepal, 2024].

Incorporating high-quality prevention, acute, and rehabilitation services has been proven to improve stroke care globally (Feigin and Owolabi, 2023). In the context of understaffed and undertrained community health system, emerging evidence highlights task-shifting, decentralization of specialized urban services into sub-urban and rural settings, and system-wide capacity strengthening as feasible strategies to address this gap in LMICs, such as Nepal (Coales et al., 2023; Ojo et al., 2021). Over a background of these systemic limitations of stroke care —this community case study details a collaborative telemedicine supported model in advancing stroke care in the region by outlining its core interventions and presents the outcomes achieved through an integrated approach.

## Methods

### Study design

This is a descriptive implementation-focused retrospective program evaluation conducted in a remote region of Nepal from August 2023 to August 2025 using a mixed-methods approach.

### Study setting

Province Hospital Surkhet serves as the primary referral center for Karnali Province, covering 10 districts of the province, as well as neighboring districts from two adjoining provinces. This 300 bedded hospital is one of the two tertiary centers of the province, serving 79,000 outpatients per year (Aref et al., 2023), and is the only center with ICU services and CT imaging available onsite. It manages an estimated 150–200 stroke cases annually, with care provided under the department of internal medicine due to the lack of on-site neurologist. Neurosurgical services are intermittent and not consistently available throughout the year.

The Nepal Stroke Project was established in 2021 as a collaboration between the Nepal Stroke Association and stroke neurologists at University Hospital Heidelberg, Germany, with the aim of strengthening access to stroke care across Nepal. The program implements a multifaceted strategy combining public awareness activities, clinical capacity building, and health system strengthening. It has contributed to national-level advocacy, including the development and adoption of the first national stroke protocol. Core activities include structured stroke care masterclasses, regular online training for physicians, technical support for establishing stroke units, and support for developing clinical stroke registries. The initiative also promotes interdisciplinary collaboration among neurologists, internists, neurosurgeons, and emergency physicians across participating hospitals, alongside in-country expert consultation for acute and complex stroke cases.

### Intervention

**Figure 1:**
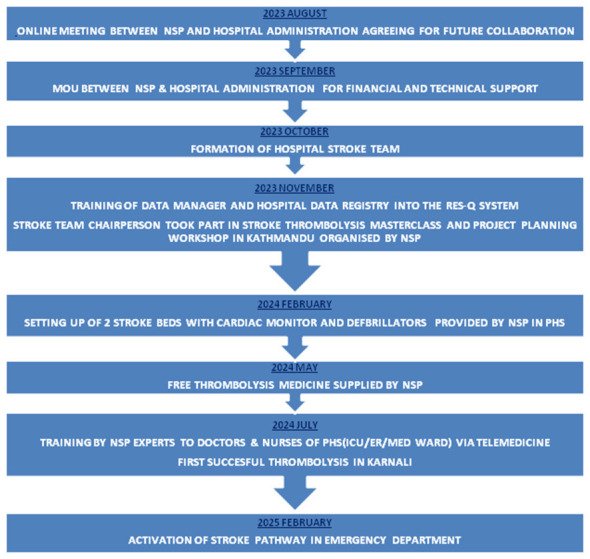
A timeline of this collaborative journey, highlighting key phases and outcomes that laid the foundation for sustainable stroke care in one of Nepal's most underserved provinces.

The implementation strategy consisted of workforce training, telemedicine enabled specialist support, workflow redesign and resource enablement. Such teleconsultation comprised more of neuroimaging consultations, deciding on need of thrombolysis, and monitoring of patients condition after thrombolysis. Remote neurologists from urban areas nationally and internationally, engaged through accessible, easy and free platforms like WhatsApp as an alternative to telemedicine consultation. They guided real-time clinical decisions, reviewed neuroimaging, facilitated and monitored patients requiring specialized health care services like thrombolysis for acute stroke patients, and handled medical issues during admission (Figure 1).

### Data collection and analysis

A structured baseline assessment and data collection protocol of the acute stroke care system at PHS was undertaken, guided by the RE-AIM framework (Glasgow et al., 2019), through retrospective review of routinely maintained hospital records, administrative documents, and internal service workflow discussions. The baseline assessment focused on infrastructure, workforce readiness, diagnostic capacity, acute stroke workflow, documentation practices and availability of thrombolysis services. For the baseline barriers in stroke care delivery, expert opinion was compiled through informal but structured discussions with frontline health care workers involved in stroke care delivery, including physician, nurses and hospital administrators, conducted during routine clinical meetings and stroke care activities.

Process indicators included stroke pathway activation, CT completion, teleconsultation, thrombolysis administration and NIHSS documentation. This assessment was used to identify service gaps and inform the subsequent implementation strategy.

The outcome indicators used to measure impact are thrombolysis uptake, stroke call activation, NIHSS documentation, ICU transfer after thrombolysis, and number of staffs trained. An internal audit of the stroke care pathway was undertaken through review of emergency records, stroke call documentation, teleconsultation logs, thrombolysis records, and ICU transfer notes to assess fidelity of implementation.

These indicators were organized according to the RE-AIM framework (Table 1).

**Table 1:** RE-AIM evaluation of the stroke service implementation model.

**RE-AIM domain**	**Indicators used**	**Early findings**
Reach	Thrombolysis	Two cases
	Number of healthcare professionals trained	10 health care professional trained
Effectiveness	Initiation of thrombolysis services	Initiated
	Teleconsultations	20 teleconsultations
Adoption	Stroke pathway activation, NIHSS documentation	Operationalized
Implementation	Workforce training	Operationalized
	Telemedicine enabled specialist support	
	Workflow redesign	
	Resource enablement	
Maintenance	Strengthening of local capacity	Stroke team formation
	Infrastructure development	Two stroke beds, bedside monitoring
	Use of existing trained manpower	Operationalized
	Routine stroke call activation	Operationalized
	Community empowerment	30 Community health workers trained

### Ethics

Administrative permission for retrospective review of institutional records and service evaluation was obtained from the hospital administration. As this was a retrospective service-evaluation and quality-improvement activity without prospective participant recruitment or additional patient-facing interventions for research purposes, formal institutional ethical review and individual patient consent were not separately sought. To protect confidentiality, only essential clinical information relevant to acute management was shared, and efforts were made to avoid transmission of direct patient identifiers, including names and other personally identifying details, wherever feasible.

## Results

### Identified baseline barriers to effective stroke care delivery

The baseline assessment revealed the following health system limitations:

- **Infrastructure gaps:** The hospital lacked dedicated stroke unit similar to other comparable hospitals in Nepal (Paudel et al., 2023). There was also no access to stroke beds or dedicated bedside equipments for the stroke patients. CT scan services were available throughout day and night, however advanced diagnostics beyond conventional CT like vascular imaging (CT angiography) or laboratory markers to guide acute stroke management were absent (Tunkl et al., 2025). There was lack of stroke-specific emergency protocol and standardized stroke triage pathway in emergency department to prioritize suspected stroke patients. Routine NIHSS documentation was not in place, and no dedicated thrombolysis service existed at baseline. Specialist consultation for acute stroke, either on site or via remote consultation was absent. In addition, dedicated stroke beds and essential monitoring equipment for acute post-thrombolysis care were not available. Review of existing workflow processes further highlighted the absence of standardized documentation of stroke cases, key time-based process indicators, including door-to-needle time and outcomes of the admitted patients. The absence of reliable data impeded quality improvement, resource planning, and evidence-based decision-making.
- **Human resource constraints:** There was also a critical shortage of specialized human resources compounding the problem, as there were no neurologists and no formally trained stroke teams within the province (Thapa, 2021). Frontline healthcare workers—primarily paramedics and general nurses—often lacked stroke-specific training, resulting in poor recognition of stroke symptoms and inadequate emergency response capabilities.
- **Financial and accessibility barriers:** These structural and workforce limitations were further exacerbated by financial barriers. One example is the high out-of-pocket cost of thrombolysis, approximating to $800 USD per treatment was unaffordable for the majority of the population (Aref et al., 2023), considering Nepal's per capita income of only $121 USD per month ($1448 USD annually; World Bank Open Data, 2025). Poor insurance coverage [Ministry of Health and Population (MoHP), Government of Nepal, 2024], lack of thrombolytic medication within the insurance package (Health Insurance Board, 2026), and perception of poor functional outcome of stroke limited out of the pocket expenditure in such setting.

### Reach—early quantitative outcomes

Early quantitative outcomes demonstrated encouraging service-level impact following implementation of the stroke care model. Annual stroke admissions increased from 154 cases per year during the pre-implementation period to 178 cases per year after implementation, reflecting improved case identification and pathway utilization. In addition, a total of 10 staff members were trained as part of the program, including three doctors, three nurses, three paramedical staff, and one administrative personnel, supporting workforce strengthening and pathway adoption.

### Effectiveness: the first cases of thrombolysis

Intravenous thrombolysis, which was not available prior to the intervention, was successfully administered to two patients. Both patients were shifted to ICU after the intervention. Over a period of 3 months, 20 stroke patients received telemedicine advice through 10 health care workers. For those patients needing specialized services like thrombolysis, multiple tele-consultations, median four per patient (range 3–5), were done per patients.

### Adoption

Although a dedicated stroke call log was not systematically maintained, review of emergency and thrombolysis records indicated that stroke pathway activation and telemedicine supported decision making was undertaken in all patients who received thrombolysis within the 4.5-h therapeutic window, as this formed an essential step of the established workflow. Service readiness was further enhanced through the establishment of two dedicated stroke beds and provision of essential monitoring equipment, including a defibrillator. NIHSS documentation was present in approximately 50% of post-implementation stroke cases, compared with no routine documentation at baseline. Door-to-CT time could not be formally assessed because time-stamped imaging records were not consistently available.

### Implementation: leveraging telemedicine- bridging specialist gaps through technology

To address the severe shortage of neurologists, guide decision making in acute care, and foster confidence in working medical officers and specialists, telemedicine became a cornerstone of the program. Over a period of 6–9 months, the hospital stroke care team transitioned to independently managing stroke cases following a structured stepwise mentorship. Ten local Health care professionals received on-the-job training with areas such as acute stroke assessment, protocol-based management, and initiation of tele-stroke consultations. Teleconsulation support still continues for complex cases and quality assurance, indicating successful capacity transfer and sustainability. Two successful thrombolysis were conducted over a period of 6 months after initiating the collaboration.

### Maintenance

**Strengthening local capacity- building a multidisciplinary stroke team:** The collaboration prioritized the formation and training of Karnali Province's first dedicated multidisciplinary stroke team involving representative specialists from department of internal medicine, emergency, radiology, nursing incharge, nursing team and hospital administration. It was a key programmatic strategy to improve stroke care delivery by coordinating clinical decision making, regular case-based discussions and facilitating time shifting among available staff. Through a combination of virtual and in-person sessions initiated in 2023, 10 frontline staff including doctors, nurses and health care workers from emergency, ICU, and medical wards were equipped with essential stroke management knowledge in two sessions of 1 h each, mentored by specialist from Nepal Stroke Project, marking a crucial step toward system-wide readiness. The training was targeted in specific areas of acute stroke recognition, NIHSS assessment, and thrombolysis workflow.

**Infrastructure development: establishing stroke-ready facilities:** Two specialized stroke beds with continuous cardiac monitoring and critical emergency equipment were introduced. This infrastructure coupled with sensitization of existing nursing and paramedic health care personnel in stroke care via targeted short terms training advanced the quality of acute stroke care. In the absence of resources for establishing stroke center, this served as a pragmatic strategy to improve stroke care delivery.

**Improving access to essential medicines—removing financial barriers:** The provision of free thrombolytic medication to Province Hospital eliminated critical cost barriers, making life-saving treatment accessible to eligible patients. Sustaining the availability of free thrombolysis medication in the long run was unrealistic and not feasible, however initial provision of costly medicine was pivotal in initiating thrombolytic therapy. It was followed by a consistent supply of medication by the hospital administration enabling uninterrupted availability of the medicine.

**Community empowerment—engaging female health volunteers for stroke awareness:** The project extended beyond hospital premises by conducting targeted awareness sessions with Female Community Health Volunteers (FCHVs)—empowering them to educate families on stroke prevention, recognize warning signs, and encouraging early healthcare-seeking behavior at the community level. Two such sessions were conducted for 30–40 FCHVS in each session with a focus on symptom recognition and appropriate acute response.

## Discussion

The collaboration between Province Hospital Surkhet (PHS) and the Nepal Stroke Project (NSP) stands out as a model for delivering quality stroke care in resource-limited settings by optimizing existing resources, addressing gaps in specialist manpower and infrastructure through tele-consultation, targeted online and onsite training, and structured capacity building. Beyond clinical improvements, the collaboration empowered local health systems and offered practical and scalable solution for advancing stroke care in other provinces in Nepal and underserved regions globally. In the absence of a structured telemedicine platform in a resource-limited setting, this implementation model demonstrates a pragmatic approach to delivering stroke care through telemedicine-guided remote assessment and management. Beyond facilitating clinical decision-making for acute stroke, the model also contributed to system-level strengthening and local capacity building by supporting protocol development, skill enhancement, and coordinated stroke care delivery in a remote context. Significant progress was achieved by leveraging available infrastructure and human resources, without requiring additional staff or costly infrastructure upgrades. Existing physicians, nurses, and paramedics were trained to deliver essential stroke care, including thrombolysis initiation.

- **Adoption:** Adoption of the implementation model was reflected in routine use of stroke call activation and increased uptake of NIHSS documentation. The consistent activation of the pathway by frontline teams suggests improved provider confidence and supports good early fidelity to the adopted workflow. In addition, the willingness to initiate thrombolysis under telemedicine guidance reflects favorable early adoption and acceptability of the model. Early engagement of hospital leadership and frontline staff helped in continuity and reduced resistance in adopting new stroke care models. However, constraints related to documentation systems and workflow integration limited the routine capture of door-to-CT time during the early phase of pathway adoption.
- **Implementation:** Fidelity of implementation was demonstrated through consistent pathway activation among thrombolysed cases, improved NIHSS documentation, and structured ICU transfer after thrombolysis. Feasibility was supported by the availability of CT imaging, stable telecommunication connectivity, and use of existing trained workforce. Following activation of the stroke call, CT imaging could be performed rapidly through active coordination by the internal medicine team and trained medical officers. Teleconsultation was led by the internal medicine specialist, while thrombolysis administration was carried out by the emergency team before transfer of the patient to the intensive care unit. The minimal operational cost of telemedicine further enhanced feasibility in this resource-limited setting.
- **Sustainability:** Empowering non-specialist providers and integrating stroke protocols, pathways, and documentation processes into routine hospital workflows enhanced sustainability, minimized disruption, and improved system efficiency. Despite its strengths, the model has several issues related to sustainability. It remains partly dependent on external project support for thrombolytic medication, telemedicine-enabled specialist input, and infrastructure strengthening, which may challenge long-term independent sustainability. Continuity of the model also depends on retention of trained emergency and internal medicine staff, and staff turnover may affect pathway fidelity. In addition, the current system has limited embedded audit mechanisms for real-time process indicators such as door-to-needle time. While the addition of two dedicated stroke beds improved acute care readiness, bed capacity may remain insufficient during high patient-volume periods. Furthermore, expansion of structured post-acute rehabilitation pathways would strengthen the comprehensiveness of the model.

### Global parallels: lessons from low-resource stroke care models

The experiences from neighboring regions further reinforce the feasibility of scalable stroke care in low-resource settings. The Tezpur Stroke Unit in North-East India demonstrated that protocol-based stroke management can be delivered by non-specialist physicians with structured, remote training via platforms like Skype and the Himachal Pradesh smartphone-enabled telestroke network highlighted how basic mobile technology like whatsApp can assist in remote decision-making and administering thrombolysis by non-neurologist under remote guidance (John et al., 2021; Sharma et al., 2016). This approach is highly cost-effective, bypassing expensive telemedicine platforms while ensuring timely care through ongoing and real time mentorship. The reliance on WhatsApp-based teleconsultation for rapid specialist input may raise concerns regarding data security, record retention, and patient confidentiality. To minimize these risks, only essential clinical information required for acute decision-making was shared, with efforts made to avoid disclosure of direct patient identifiers wherever feasible. Nevertheless, the use of general-purpose messaging platforms remains a limitation of the model and highlights the need for secure, institutionally approved telemedicine platforms in future scale-up phases. Collectively, these models, together with our own experience, further substantiate the feasibility and effectiveness of such context-adapted strategies and highlight their significant potential for sustaining and scaling stroke care delivery in resource-constrained settings.

This collaboration was successful due to these three main points—committed multidisciplinary stroke team, and use of standardized stroke care protocols, and use of simple digital health technology. Strengthened community awareness through engagement of female community health volunteers also supported this collaboration.

### Limitations of the study

This study was a descriptive, implementation-focused program evaluation based on retrospective review of routinely maintained institutional records and internal audit documents. As such, it was not designed as a prospective research study with predefined outcome measures, which limits the ability to draw causal inferences regarding the direct impact of the intervention on patient outcomes. The number of cases included in the post-implementation evaluation was relatively small, reflecting the early phase of service establishment and the case volume at a single provincial referral hospital. In addition, the absence of a formal stroke registry or a prospectively designed systematic data collection protocol limited the completeness and consistency of available data. Several key process and clinical indicators were derived retrospectively from routine records, which may be subject to documentation variability and incomplete capture. Formal evaluation of patient-level clinical outcomes, including in-hospital mortality, functional recovery, door-to-needle time, discharge modified Rankin Scale status, and long-term neurological outcomes, was not systematically performed within the scope of this service evaluation. A formal thrombolysis rate based on clinically eligible cases could not be determined because denominator data for time-window eligibility and contraindications were not routinely available. The follow-up period was limited to the early implementation phase of the program, which restricts assessment of long-term sustainability, durability of workforce training effects, and patient outcomes beyond the immediate hospitalization period.

### Remaining challenges and future considerations

A key challenge relates to long-term sustainability of the model beyond the initial implementation period. While telemedicine itself requires minimal operational cost and the pathway has been integrated into existing workforce structures, continued access to thrombolytic medication and securing long-term voluntary remote specialist support remain dependent on institutional and governmental ownership. Future strengthening should therefore focus on transition from externally supported inputs to hospital- and policy-level integration, including dedicated financing, secure telemedicine platforms, and routine prospective outcome monitoring. Other broader challenges of a low resource setting like poor community awareness of stroke symptoms and the critical time window for intervention, delayed care-seeking behavior, absence of advanced interventions like thrombectomy, high turnover of trained personnel and limited post-stroke rehabilitation services also exist. Integration of comprehensive stroke care into existing healthcare infrastructure is vital to improving long-term outcomes and functional recovery for stroke survivors. Drawing inspiration from the Nepal Stroke Project's efforts, funding has been successfully secured from the World Stroke Organization to implement a context-sensitive, video-based stroke prevention initiative. This project will focus on interactive, locally adapted educational materials designed to enhance community-level awareness and reduce stroke incidence at its root.

Based on the findings, we recommend that sustaining and scaling stroke care in resource-limited settings like Karnali requires strong policy integration, with formal recognition of stroke units, standardized acute care protocols, and essential medicines such as thrombolytics within provincial and national health priorities, alongside financial protection mechanisms to ensure equitable access. Complementary investments in workforce capacity, structured telestroke networks, and community-based prevention strategies are essential to reinforce system resilience and long-term impact.

## Conclusion

The collaborative model between the Nepal Stroke Project (NSP) and Province Hospital Surkhet (PHS) exemplifies that substantial progress in stroke care is possible even in rural and remote health care setting. Through the establishment of a dedicated stroke team, setup of stroke beds, successful initiation of thrombolysis via telemedicine network, and targeted capacity building of healthcare personnel, this initiative has laid the groundwork for sustainable, life-saving stroke care in Karnali. This collaborative model can be systematically integrated into national, regional and international stroke policy frameworks.

## Data Availability

The original contributions presented in the study are included in the article/supplementary material, further inquiries can be directed to the corresponding author.
